# State of the Art of Natural Killer Cell Imaging: A Systematic Review

**DOI:** 10.3390/cancers11070967

**Published:** 2019-07-09

**Authors:** Michela Varani, Sveva Auletta, Alberto Signore, Filippo Galli

**Affiliations:** Nuclear Medicine Unit, Department of Medical-Surgical Sciences and of Translational Medicine, Faculty of Medicine and Psychology, “Sapienza” University of Rome, 00161 Rome, Italy

**Keywords:** NK cells, immunotherapy, molecular imaging, QUADAS-2 analysis, therapy evaluation

## Abstract

Natural killer (NK) cell therapy is a promising alternative to conventional T cell-based treatments, although there is a lack of diagnostic tools to predict and evaluate therapeutic outcomes. Molecular imaging can offer several approaches to non-invasively address this issue. In this study, we systematically reviewed the literature to evaluate the state of the art of NK cell imaging and its translational potential. PubMed and Scopus databases were searched for published articles on the imaging of NK cells in humans and preclinical models. Study quality was evaluated following Quality Assessment of Diagnostic Accuracy Studies (QUADAS-2) criteria. We pooled studies as follows: Optical, magnetic resonance imaging (MRI) and nuclear medicine imaging with a total of 21 studies (*n* = 5, *n* = 8 and *n* = 8, respectively). Considering the limitation of comparing different imaging modalities, it appears that optical imaging (OI) of NK cells is very useful in a preclinical setting, but has the least translational potential. MRI provides high quality images without ionizing radiations with lower sensitivity. Nuclear medicine is the only imaging technique that has been applied in humans (four papers), but results were not outstanding due to a limited number of enrolled patients. At present, no technique emerged as superior over the others and more standardization is required in conducting human and animal studies.

## 1. Introduction

Recent advances in immunotherapy highlighted how our immune system can be efficient in fighting cancer. Indeed, the combination of innate and adaptive immunity is able to kill abnormal cells, as well as exogenous microorganisms. Indeed, several studies have shown that immunodeficiency, due to genetic defects or external causes such as HIV, can lead to an increase in tumor incidence [1]. Like in other biological systems in which the prey evolves to escape the predator, cancer cells can evade the immune response. They can evolve by losing antigens, immunogenicity or create an immunosuppressive microenvironment [2]. Different strategies such as monoclonal antibodies (mAb), checkpoint inhibitors, adoptive cell transfer (ACT), vaccines and other drugs can be used to revert evasion mechanisms and allow immune cells from the host to infiltrate lesions to exert their cytotoxic activity [3]. The term immunotherapy includes all the above-mentioned approaches and gives new hope to patients with metastatic advanced cancers.

The immune response is mediated not only by T lymphocytes but also by other subpopulations like B lymphocytes, dendritic cells (DC), macrophages and natural killer cells (NK). T and B cells recognize exogenous antigens and mediate a specific response, with an effective memory to protect the body by future attacks. DC, macrophages, monocytes and NK cells mediate a rapid and temporary response releasing effector molecules, such as perforin/granzymes B, to lyse cancer cells. In particular, NK cells that were identified as lymphoid cells with lytic activity against tumor cells play a relevant role in cancer cells detection and destruction without prior immunization [4,5]. Several studies reported how a high or low infiltration of NK cells in a cancer microenvironment can influence patient prognosis. Coca et al. [6] has proven in patients with colorectal cancer and Ishigami et al. [7] in patients with gastric carcinoma that the NK infiltration into the tumor can influence its growth. Infiltration of NK in the intratumoral section was positively correlated with a better response against the tumor and consequently an increase in survival [8]. Similarly, patients with higher levels of NK showed less metastatic dissemination in lymph nodes, if compared with patients with lower levels of NK infiltration that showed a worse survival curve. Vaquer et al. [9] also evaluated the NK activity in the peripheral blood of women with squamous cell carcinoma of the uterine cervix, demonstrating that lower levels of NK cells corresponded to a higher disease dissemination. On the basis of these results, it is clear that NK-cell based immunotherapies offer great potential as alternative therapeutic approaches in oncological patients [10].

NK cell immunotherapy offers many advantages over T cell immunotherapy. Its safety is particularly relevant, since neither graft-versus-host disease (GVHD) nor cytokine release syndrome have been observed [11]. Moreover, the immunotherapy outcome is potentially unrelated to tumor dedifferentiation status because of the self/non-self-ligand recognition mechanism by NK cells. In fact, monoclonal antibodies (mAbs) epitope-based vaccines with highly specific mAbs might not work due to loss of specific antigens by cancer cells. Like T cells, NKs can be collected from human blood and cultured ex vivo, for adoptive cell therapies, but do not require a suicide vector when genetically unaltered [12]. To date different therapeutic strategies have been described [13]. NK cells can be stimulated with systemic administration of activating cytokines, like interleukin-2 (IL2), as described in patients with renal cell carcinoma and metastatic melanoma [14,15]. Due to the systemic toxicity caused by IL2, researchers evaluated the possibility to expand and activate NK cells ex vivo before their reinfusion into the patient. This led to increased therapeutic effect with limited adverse events [16]. The use of haplo-identical allogeneic NK cells, ex vivo also expanded and injected in patients goes beyond the limit of autologous NK cells subjected to inhibitory signals recognizing self-major histocompatibility complex (MHC) on tumor cells. The killer-cell immunoglobulin-like receptor (KIR) mismatch of cytokine activated NK cells has demonstrated an antitumor effect in metastatic melanoma, renal cell carcinoma, Hodgkin’s disease and others [17,18,19]. Limitation of this approach could be the contamination with other immune cells, like T cells during withdrawal with possible rejection for MHC mismatch, with GVHD [20,21]. Several studies have been performed to increase the cytotoxic capacity of allogeneic/autologous NK cells expanded ex vivo, to test the stimulation with different cytokines, the co-culture with autologous accessory cells and with autologous/allogeneic feeder cells [22].

The activation of NK cells cytotoxic functions, after the binding between the FcγRIIIA receptor and the Fc-antibody, was evaluated as a potential immunotherapeutic approach. Therefore, it has been demonstrated how mAbs, as rituximab, cetuximab and trastuzumab, induce an increase of NK-mediated antibody-dependent cellular cytotoxicity (ADCC) and can be used to treat some type of tumors [23]. NK cells can be also collected from patients with lymphoma/leukemia or from hematopoietic precursor cells, like embryonic or induced pluripotent stem cell (iPSC) [24,25]. These immortalized NK cell lines can be cultured in vitro and have a high anti-tumor activity if compared to autologous/allogeneic NKs. They are easy to expand ex vivo, good manufacturing practices (GMP) compliant and they can be modified to increase their cytotoxicity [26,27,28]. The most used NK cell lines are the NK-92, isolated in 1992 from a patient with a non-Hodgkin’s granular lymphoma and with a CD56^+^CD2^+^CD57^+^CD3^–^ phenotype. These cells depend on IL2 for growth and showed a high cytotoxicity related to both an elevated expression of activated receptors, like NKp30, NKp46, 2B4, NKG2D and CD28 and production of molecules with lytic function [29]. However, before infusion in patients, these cells require irradiation to prevent in vivo uncontrolled proliferation, but they are still able to produce cytokines and kill their target upon recognition of specific markers [30]. Modification of specific genetic aspects can modulate their specificity against malignant cells or increase their effector functions [31]. Kim et al. demonstrated that it is also possible to modify the tumor phenotype, (i.e., by I-131 pre-treatment) to increase NK cell infiltration in the lesions with therapeutic effects [32]. Recently, very promising results were obtained using chimeric antigen receptor NK cell-therapies that showed a safe profile and enhanced specificity against tumor cells if compared to chimeric antigen receptor (CAR)-T cells [33,34].

### NK Imaging: Implications for Immunotherapy

Conventional response criteria, like Response Evaluation Criteria in Solid Tumors (RECIST), were established for patients treated with chemotherapies [35]. They still remain the gold standard, but present several limitations when applied to patients undergoing immunotherapies. Indeed, there is the risk of misinterpretation in those patients with transient tumor enlargement due to infiltration of immune cells (pseudo-progression) or detection of new lesions followed by response to therapy. In these patients [^18^F]FDG-PET/CT is not useful and new biomarkers and diagnostic strategies are needed [36,37]. This lack of diagnostic tools to predict and follow-up therapy efficacy in T cell-based immunotherapies has been extensively discussed elsewhere and new diagnostic criteria are under investigation [38]. This very same limitation also applies to NK-cell based therapies and many efforts have been made to develop new tools in the molecular imaging field [39]. To this purpose many imaging techniques have been explored with limited success. Direct and indirect approaches have been attempted to label cells with fluorophores, contrast agents or radioactive isotopes [40]. Direct labeling is achieved by collecting NK cells from a donor, ex vivo labeling and reinjection in the same subject. This reduces the signal from background and the labeling procedure is mostly simple and based on incubation, without the need of genetic manipulation of cells.

However, leakage of most labels from the cells and release of the label from dying cells is often observed. A common limitation is also represented by the dilution of the intracellular label as cells divide. Since it is not possible to add new labeling agents after cell reinfusion, imaging at very late time point is not always feasible [41]. 

Indirect labeling requires a genetic approach, based on the insertion of a reporter gene that encodes for specific enzymes or receptors for a given labeling agent. This approach is very specific and does not suffer from dilution effects allowing long-term visualization of NK cells. However, there is the need to genetically engineer cells and this requires specific infrastructure, facilities and expertise, therefore it is still limited to pre-clinical research. Another strategy is based on the use of a labeling agent able to bind to a specific antigen on the cell surface of target cells in vivo. The molecule can be either an antibody or a peptide, labeled with a reporter agent, without the need of any ex vivo manipulation [42]. The drawback is represented by unspecific uptake by healthy tissues (mainly liver and/or kidneys) due to physiologic biodistribution of the labeling agent.

In this scenario, optical imaging (OI), magnetic resonance (MR) and nuclear medicine (NM) are the techniques with the highest translational potential and have different pros and cons listed in Table 1.

Optical imaging is a radiation-free and low-cost method to visualize ex/in vivo NK cells with high sensitivity and specificity, especially useful in preclinical studies with small animals. Optical techniques exploit the photons of the light to visualize the target of interest by employing exogenous or endogenous molecules and reporter genes [43]. Among the OI techniques, the in vivo bioluminescence imaging (BLI) is a non-invasive method to study biological processes in vivo. It is based on a molecular reporter expressing an enzyme (luciferase) that mediates the oxidation of a molecular substrate with light emission [44,45]. Despite its good resolution, it is characterized by low tissue penetration (1 mm for fluorescence, 3 mm for bioluminescence) that limits its use in humans [46].

It is possible to partially overcome this limitation by using in fluorescence, the near-infrared (NIR) dyes that emits between 650 and 900 nm [47]. In this window, light absorption by hemoglobin is very low resulting in very low background together with low auto-fluorescence. A very useful pre-clinical technique is intravital microscopy (IVM) that comprises several microscopy techniques to monitor and eventually quantify in vivo a specific target labeled with a fluorophore [48]. The choice of the technique depends on the depth of the organ of interest, due to tissue attenuation that prevents the dynamic diffusion of light. If the target is 50–100 µm in depth, the best choice can be multi-photon microscopy (MPM) [49]. This method is able to absorb simultaneously two or more photons with a wavelength from 800 nm to 2500 nm (near-infrared) or from 700 nm to 1000 nm (infrared). Compared with other microscopy techniques, in particular with spinning disk confocal microscopy, MPM needs a high concentration of photons in a small area, reducing photobleaching and phototoxicity [50]. MPM has been very useful to investigate the biological process of NK cells, in particular their interactions with DC cells. Some studies revealed the necessity of a direct contact between NK and DC cells for their activation with short and dynamic interactions [51,52].

MR imaging provides anatomical and physiological information with a high resolution (10–100 μm). MRI contrast agents have an unlimited deep penetration with a good contrast of soft tissue, but their limitation consists in a low sensitivity [53]. The contrast agents can be T2-negative, with a reduction of signal, or T1-positive, with an increase in signal. The main negative contrast agents are based on superparamagnetic iron-oxide nanoparticles (SPION) with an iron oxide core with very high T2 relaxivities, up to 200 (mMs)^−1^ [54].

Nuclear medicine imaging relies on the use of radiopharmaceuticals and overcomes limitations of OI like light diffusion in the human body [55]. Radiopharmaceuticals like ^111^In-oxine and ^99m^Tc-HMPAO are currently used to radiolabel white blood cells for infection/inflammation imaging. The same approach has been applied to many other immune cell subtypes with limited success. It allows functional visualization of molecular and cellular processes with a good spatial resolution and high sensitivity [56,57].

Despite the potential of these imaging techniques and the attempts to label NK cells, we are still far from the development of a diagnostic tool to be used in clinical practice. This may allow physicians to select the best therapeutic option for a specific patient and to monitor therapy outcomes to promptly take corrective actions, if needed [58]. Moreover, it may have an impact in a preclinical setting for the discovery and testing of new drugs, reducing sample size, refining obtained data and saving time and money. In the present work, we systematically reviewed the literature to evaluate the current status of approaches with the most translational potential.

## 2. Materials and Methods

### 2.1. Inclusion Criteria

In the present review, only published articles that met the following criteria were included: Articles (only original papers); labeling compound (fluorophores, contrast agents, radioisotopes/radiopharmaceuticals); target (NK cells); translational imaging technique (OI, MRI, NM). Publications selected for this review were from peer-reviewed indexed journals only.

### 2.2. Exclusion Criteria

Reviews, case reports, abstracts, editorials, poster presentations, publications in languages other than English, studies in which compounds were not essentially specific for NK cells (e.g., natural killer T (NKT) cells), articles with limited translational potential and/or human applications (e.g., microscopy studies, IVM, bioluminescence), were excluded. The decision to include or exclude an article was made by consensus.

### 2.3. Search Methods for Identification of Studies

The selection of published articles was based on the Preferred Reporting Items for Systematic Reviews and Meta-Analyses (PRISMA) guidelines [59]. We selected all studies published in English language regardless of publication status (published, ahead of print, in press, etc.). The following algorithm was used: (Imaging OR tracking) AND (NK or natural killer) AND (nuclear medicine OR MRI OR magnetic resonance OR radioactivity OR optical imaging OR fluorescence). We searched for published papers present in PubMed and Scopus. The literature search was broadened to all reference lists of all retrieved articles.

### 2.4. Data Extraction and Management

All authors screened independently full-text manuscripts for eligibility, reporting essential data in a summary table. Risk assessment of any potential bias and data collection was performed by using a standardized questionnaire, adjusted to included studies. Any disagreement was resolved through discussion.

### 2.5. Risk Assessment of Bias in Included Studies

The included studies were evaluated by using the Quality Assessment of Diagnostic Accuracy Studies (QUADAS-2) approach for any potential bias [60]. For human/animal studies we evaluated: i) Patient/animal selection (inclusion/exclusion criteria), ii) index test (experimental design, labeling method), iii) reference standard (positive/negative controls), flow and timing and applicability concerns (Table A1).

Due to heterogeneity of included studies (animal models, labeling compounds, etc.), we classified studies in relation to the imaging technique (OI, MRI, NM).

## 3. Results and Discussion

### 3.1. Data Synthesis

We present here a systematic review on the state of the art of NK cell imaging to identify the most promising approaches to be translated into clinical practice among OI, MRI and NM. We performed a QUADAS-2 analysis to evaluate the strength of reported findings and guide researchers to adopt systematic approaches when performing animal experiments.

**Figure 1:**
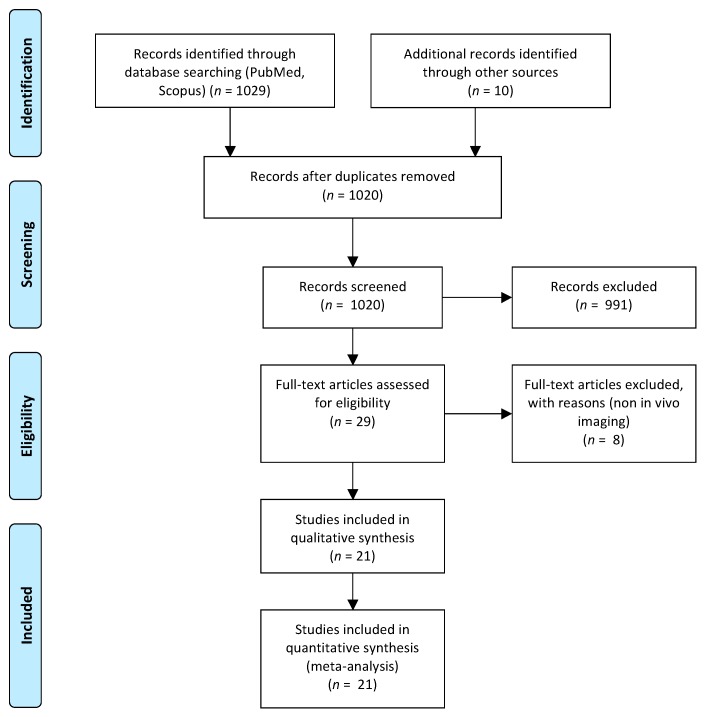
Preferred Reporting Items for Systematic Reviews and Meta-Analyses (PRISMA) flow chart of the literature search and selection process.

Due to differences between studies, data were pooled, when possible. When not, they were separately described. We found 29 studies that described potential imaging tools to follow NK trafficking in tumor lesions. After excluding the non-eligible articles, we analyzed 21 published studies (Figure 1). A summary of results is reported in Table 2, whereas a summary of QUADAS-2 analysis is reported in Table 3. The studies were pooled by imaging technique: OI (*n* = 5), MRI (*n* = 8) and NM (*n* = 8).

### 3.2. Optical Imaging 

Four studies described the labeling of NK cells with fluorescent dyes. In three of them, imaging was used as a tool to determine migration and therapeutic efficacy against human xenografts in animal models. This way it is possible to retrieve useful information, like the number of tumor infiltrating NK cells, in the same subject and over multiple time points without sacrificing it for immunohistochemistry or other laboratory tests. This allows researchers to reduce the number of animals and increase the quality of the results. Considering the limited number of papers in the literature, OI studies appeared to have very limited sources of bias and highlights how this technique is relatively cheap and useful, but still confined in a preclinical setting. 

An example of the use of a NIR dye, 1,19-dioctadecyl-3,3,39,39-tetramethylindodicarbocyanine (DiD), has been reported by Tavri et al. [61] Their aim was to track NK cells in a model of prostate cancer. NK-92 cells were modified through retroviral transduction to target the tumor-associated epithelial cell adhesion molecule (EpCAM) antigen, highly expressed in prostate cancers. These modified NK-92-scFv(MOC31)-zeta cells were injected in athymic rats bearing a human prostate xenograft. A control group was injected with the same amount of DiD-labeled NK-92. The animals were imaged before and 1.5, 8 and 24 h post NK cell administration. Results showed that fluorescence uptake in the tumor was significantly higher in rats that received NK-92-scFv(MOC31)-zeta cells if compared to the control group. This finding was also confirmed by fluorescence microscopy, however, not every human prostate cancer is characterized by a high expression of EpCAM antigens, so it would be necessary to confirm these results in other prostate cancer lines.

NIR fluorescent nanocrystals (QD705) were used by Lim et al. [62] to monitor the therapeutic efficacy of NK cell immunotherapy. QD705 were conjugated with an anti-human CD56 antibody and incubated with NK-92MI cells. In vitro studies were performed to evaluate the modified NK-92MI cells, avoiding sources of bias. The labeling did not show changes in cytolytic function, in interferon-γ (IFN-γ) production and in the viability of the cells. Melanoma tumor bearing mice were intratumorally injected with QD705-anti-CD56-NK-92MI or phosphate buffered saline (PBS), as a negative control, after six and 12 days from tumor implantation. NIR fluorescence imaging was performed at day 13 showing high accumulation in the tumors of mice injected with QD705-anti-CD56-NK-92MI. Ex vivo studies revealed a strong reduction of tumor volume in these mice if compared with the control group. Imaging was used with the same purpose by Lee et al. [65] that evaluated the efficacy of an NK-cell-recruiting protein-conjugated antibody (NRP-body) in primary and metastatic pancreatic duct adenocarcinoma in murine animal models. They labeled ex vivo expanded NK cells from healthy donors with 1,1′-Dioctadecyl-3,3,3′,3′-Tetramethylindotricarbocyanine iodide (DiR) and demonstrated increased NK infiltration into tumors with a significant anti-cancer effect. This is a key example of how NK cell imaging could improve drug discovery and efficacy evaluation in a preclinical setting. Uong et al. performed real-time tracking of NK cells in a triple-negative breast cancer mouse model [64]. NK cells were labeled with different molar concentrations of ESNF13, a NIR fluorophore previously used to monitor cell proliferation and differentiation with low cytotoxicity. They performed a preliminary biodistribution of ex vivo labeled NK cells in normal mice followed by tumor targeting studies in a human breast cancer (MDA-MB-231) xenograft model. They reported NK cell localization in the lungs at 1 post-injection (p.i.), however at 4 h p.i. fluorescence was mainly observed in the kidneys, probably due to the release of the dye in the blood pool. In tumor bearing mice, the real-time trafficking of NK cells revealed fluorescence accumulation in the lesions at 4 h p.i. Immunochemistry studies confirmed the presence of lung metastasis with high NK cell infiltration that was also observed at the tumor margin in the primary site. All these studies were properly designed and we did not report any critical source of bias. However, despite the promising results, there are some issues to take in account for possible human applications. Indeed, many fluorochromes like DiD are not approved by FDA and, in general, OI agents have a very limited use in the clinical phase. Therefore, their replacement with radioisotopes or paramagnetic molecules should be carefully considered to translate these approaches in humans.

Jang et al. [63] tried to increase cancer infiltration of NK cells loaded with iron oxide nanoparticles (Fe_3_O_4_/SiO_2_) by applying an external magnet in proximity of tumor lesions. Imaging was performed by labeling the cells with a NIR fluorescent agent and authors could demonstrate the accumulation of 2%–34% of injected NK-92 cells within 10 minutes from injection. However, after magnetic field removal the fluorescent signal quickly disappeared from the tumor region. This approach suffers from many limitations including signal specificity and low penetration of fluorescence when imaging deep tumor lesions. The lack of cancer cells origin also could have introduced biases in the study. 

### 3.3. Magnetic Resonance Imaging

Seven studies describe the use of SPION to label NK cells for MRI applications. Accumulation of labeled cells in a specific site results in the visualization of a hypointense region. Daldrup-Link et al. [69] evaluated different techniques (simple incubation, lipofection and electroporation) to label NK-92 cell lines with SPIO ferumoxides and ferucarbotran. The cells were genetically modified to express a CAR specific to the tumor-associated ErbB2 (HER2/neu) antigen. Then, NK-92-scFv(FRP5)-zeta cells were conjugated with Fe revealing a higher labeling efficiency when using ferucarbotran. Whereas the lipofection and electroporation produced an efficient labeling, simple incubation revealed a low yield. On the other hand, electroporation caused significant decrease of cell viability if compared to lipofection, which revealed a preserved viability of cells and high labeling efficiency. Mice with mammary tumors were injected with ferucarbotran-labeled NK-92 cells and NK-92-scFv(FRP5)-zeta cells. T2*-weighted AMR images showed a decreasing signal in tumors of mice injected with genetically modified NK cells, but not with normal cells. This result demonstrated an increased accumulation of NK-92-scFv(FRP5)-zeta cells in tumors and also an enhanced cytotoxic function. The QUADAS-2 analysis revealed that a hypothetical source of bias could be related to the unspecified ATCC origin of the cell line NIH 3T3. Similar results were obtained by Meier et al. [66] that compared ferumoxides SPION-labeled engineered NK-92 cells, expressing EpCAM-specific CARs, with parental NK-92 cells. Since EpCAM antigens are expressed on prostate cancer they selected a rat xenograft model bearing a DU145 tumor. In this study, SPIO labeled NK-92 and NK-92-scFv(MOC31)-z cells were injected in the tail vain of rats and MRI was performed at 1 h and 24 h p.i. Results showed a significant decrease in the tumor contrast-to-noise ratio with SPIO-labeled NK-92-scFv(MOC31)-z cells but not with SPIO-labeled parental NK-92 cells. This result was also confirmed by histopathology and represents a possible translational application of MRI in humans to monitor immunotherapy efficacy. We did not report any potential source of bias in this study. The SPIONs labeled NK-cells were also tested by Sheu et al. with an intra-hepatic-arterial (IHA) infusion in hepatocellular carcinoma (HCC) bearing rats [68]. The choice of this administration route resulted in a higher dose and homing of NK cells in the tumor, due to the enhanced blood supply via hepatic artery in liver cancers. After MRI T2* measurements it was possible to image and quantify labeled NK cells after infusion. The T2* values were compared between normal liver and tumor, before and after infusion, but not with another route of administration, such as the intra-venous (i.v.), leading to a possible source of bias. After injection the T2* values decreased both in normal and cancer tissue, but with the decrease in tumors was higher. The time flow between NK injection and MRI studies was not reported, introducing another possible bias by QUADAS-2 analysis.

Su et al. [73] studied the same route of administration in the same rodent model using NK cells labeled with FDA-approved SPIONs (Feraheme®). Labeled cells were injected through trans-catheter IHA infusion and MRI was performed at 24 h, 48 h and 8 days p.i. They used two control groups to avoid sources of bias, such as IHA infusion of saline and i.v. infusion of NK cells. They also analyzed the tumor volume, evaluating any change after NK cell administration. In the three groups, the T2* values of tumor volume before NK cell injection were without significant differences, preventing a source of bias. Significant values of R2* was measured between baseline and 24/48 h post-IHA infusion and correlated with a strong therapeutic response in the following eight days after NK infusion. The MRI data correlated with histological studies, confirming the inhibition of tumor growth when using NK based adoptive transfer immunotherapy. 

Similarly, an intraportal vein trans-catheter infusion (i.p.v.) was used by Li et al. [70] to deliver NK cells in a HCC tumor. They used FDA-approved drugs (heparin, protamine and ferumoxytol-HPF) to obtain ultrasmall SPION labeled-NK cells for therapeutic and imaging purposes. This administration route has been investigated for treatment of small nodules in a situation of hepatic cirrhosis. MRI imaging was performed before, 30 min and 12 h p.i. in tumor bearing rats. T2*W data showed a high signal intensity before and 30 min after injection of SPION-labeled cells, whereas at 12 h p.i. the signal became more heterogeneous. The lack of a late time point could be to a potential source of bias in the QUADAS-2 analysis. The authors raised the same issue and anticipated the need of later imaging time points to predict a longitudinal response during adoptive immunotherapy [82]. Several administration routes, such as i.v., intraperitoneal (i.p.) and subcutaneous (s.c.), have also been investigated by Mallett et al. [67] for immunotherapy with a novel NK cell line (KHYG-1) labeled with SPIONs (MoldayION Rhodamine B). These cells were selected by the researchers for their higher in vitro cytotoxic potential against cancer cells, if compared with NK-92 cell lines. MRI and histology confirmed KHYG-1 accumulation in cancer sites of murine models after a s.c. injection. Migration of KHYG-1 after i.p. and i.v. injection was revealed only by histology but not detected by MRI. This is probably because of the low iron content per cell or low infiltration of KHYG-1 cells in tumor. Therefore, it might be desirable to optimize the cell labeling protocol with different methods, like electroporation or transfection, already used to label NK-92 cells. This could have avoided any source of bias. In this study, a reduction of tumor volume after NK cell administration was not reported, probably because of a relatively large size at the beginning of the treatment or because of the absence of IL2 supplement to maintain KHYG-1 activation, which might represent potential sources of bias.

In the last years, fluorine-19 (^19^F) emerged as a novel agent to label cells for MRI applications in immunotherapies. Bouchlaka et al. [72] and Somanchi et al. [71] used this non-radioactive isotope to label NK cells and evaluate their biodistribution, homing and therapeutic efficacy by MRI. In vitro studies showed that ^19^F-labeling does not alter the NK cell characteristics, such as viability, cytotoxicity or expression of cytotoxic receptors. They were the first to explore the possibility to visualize ^19^F-labeled NK cells infiltrating tumors by MRI. In vivo imaging of ^19^F-NK cells was observed for s.c./intratumoral administration routes, whereas it was not achievable after with i.v. injection, that unfortunately is the main administration route for therapeutic approaches. This is a significant limitation for feasibility of this approach in humans. The possible death of NK cells and fluorine-19 phagocytosis by macrophages or DC also can lead to false positive signals, therefore more studies are needed to address these issues. The lack of an adequate reference standard is the only potential source of bias in the study by Somanchi et al.

### 3.4. Nuclear Medicine Imaging

A total of eight studies reported the use of radiopharmaceuticals as labeling agents to follow in vivo trafficking of NK cells. Studies were sorted by imaging modality: SPECT and PET. 

Radiolabeled white blood cell scintigraphy is a well-established technique for imaging of inflammation and infection. Leukocytes are easily labeled ex vivo with either ^111^In-oxine or ^99m^Tc-HMPAO that are able to passively diffuse through the plasma membrane. Therefore, first attempts in humans with ^111^In-oxine-NK cells were reported in 1990 by Hercend et al. [74]. Their primary aim was to evaluate the efficacy of IL2-activated NK cells with contemporary IL2 infusions in patients with metastatic renal carcinoma. In addition, they also injected radiolabeled NK to evaluate their biodistribution and infiltration of metastatic lesions. Patients were enrolled following well-defined inclusion criteria: i) Histological confirmation of metastatic renal cell cancer, ii) Karnovsky index above 70% and iii) presence of serum IgG antibodies direct for the Epstein–Barr virus (EBV). Twelve patients were initially included and nine were infused with IL2 activated-NK cells. However, ^111^In-oxine-NK scintigraphy was performed in only two out of twelve patients, leading to a high risk of bias for an accurate evaluation of NK cells biodistribution. Indeed, no radioactive uptake at tumor sites was observed. Given the long half-life of indium-111 the time points were also not accurately defined. At 6 h, radioactivity was confined in the lungs, whereas at 48 h and 72 h it moved to the liver, spleen and bone. Since indium-111 has a relative long half-life (67.2 h), imaging at a late time point can be performed with increased target-to-background ratios. This has been performed by Brand et al. [75] that acquired scintigraphic images at 144 h p.i. to evaluate organ biodistribution and clearance of allogeneic NK cells in patients with renal cell carcinoma and metastases in lungs and/or liver. Six patients were enrolled with specific criteria (Karnowski Index >3; progressive disease confirmed by computed tomography (CT) scan; no therapeutic possibilities) but a scintigraphy was performed in only three patients, leading to a potential source of bias. Authors confirmed that at 2 h p.i. NK cells distribute in the lungs, but at 144 h they migrate to the liver, spleen and bone marrow. Retention of radioactivity was observed for up to five days revealing either survival of radiolabeled cells or phagocytosis from the reticulo-endothelial system. Scintigraphic images were compared with CT scans with controversial results. Four metastases were detected by CT, but only two were observed in scintigraphic images. The other two lesions showed very low or no activity, probably due to necrosis caused by radiotherapy.

The same group followed this study up by the addition of [^18^F]FDG-PET/CT as a reference standard to reduce any risk bias [76]. Small tumor lesions and large necrotic metastases did not show any uptake of radiolabeled NK cells, whereas lesions with an elevated glucose metabolism did. Matera and colleagues [77] studied the differences in administration route of ^111^In-oxine-NK cells: In i.v. vs. intra-arterial (i.a.) injection in three patients with liver metastases from a primary colon carcinoma. Subjects were i.a. injected with ^111^In-oxine-NK and then, after 30 days, the same radiopharmaceutical was injected i.v. The resulting scintigraphic images showed that, at 6 h p.i., the signal from the lungs was higher for the i.v. administration route. I.a. administered NK cells mainly migrated into the spleen and liver with persistence for up to 96 h. The comparison of liver images after the two different radiopharmaceutical injection modalities at 6 h showed that the migration of NK cells in metastases was clearly visible with i.a. compared with the i.v. administration. The results supported the i.a. route for NK administration with a significant accumulation in tumor metastases. Technetium-99m labeled-phytate was injected i.v. as a standard to evaluate the extent of normal liver parenchyma, before the ^111^In-oxine-NK administration.

Only one study reported the use of an in vivo approach to target NK cells in vivo. For this purpose, an anti-CD56 mAb was radiolabeled with technetium-99m and studied in vitro and in animal models [78]. Human NK cells or human granulocytes as negative control were i.v. injected in SCID mice bearing an anaplastic thyroid cancer (ARO) xenograft in the right thigh. After 24 h the radiolabeled mAb was injected i.v. and γ-camera images were acquired up to additional 24 h p.i. Accumulation of radiolabeled mAb was observed in tumors of mice that received NK cell injection (target-to-background ratio (T/B) = 6.02), but not in the group that received granulocytes. This approach could overcome limitations related to the use of ex vivo labeled NK cells, such as cumbersome procedure and radiolysis due to radioisotope incorporation inside the cells [83]. However, clinical studies with a humanized mAb are needed to confirm the feasibility of this approach in humans. Unclear origin of cell line and origin of animals could be a source of bias.

The higher spatial resolution of PET could overcome the limits of SPECT imaging, especially when trying to image a very low number of tumor infiltrating immune cells.

In 1993, Melder et al. [79] explored the use of [^11^C]-methyl iodide to radiolabel cells ex vivo. They compared the biodistribution of IL2-activated and non-activated NK cells in a murine FSaII fibrosarcoma model. They observed that 4%–30% and 3%–4% of activated and naive NK accumulated in tumors. The cancer cells were injected in the lateral vein of mice to make a tumor site distant from internal organs and to create a simple access for radiopharmaceutical injection. Despite this, the unclear origin of mice and tumoral cells, and, also, the absence of negative controls and in vitro study, could lead to a high risk of bias.

In another study, they performed a comparison between [^11^C]methyl iodide- and [^18^F]FDG-labeling of NK, reporting high release of radioactivity from the cells (21% after 1 h). By contrast, [^11^C]methyl iodide-labeling was stable and showed no cytotoxicity. However, the choice of carbon-11 is a potential source of bias, since its half-life (20.3 min) does not allow acquiring images after 1 h p.i. (timing domain of QUADAS-2 analysis). Indeed, at early time points NK cells mainly accumulate in the lungs [80].

Meier et al. [81] did not report the same issues with [^18^F]FDG radiolabeling of NK-92 cell lines and the genetically modified NK-92-scFv(FRP5)-zeta cells. These cells express a chimeric antigen receptor specific for human epidermal growth factor receptor 2 (HER2) expressed by some types of cancer. The two radiopharmaceuticals were administrated in mice with HER2/neu-positive NIH/3T3 tumors, and images acquired at 1 h p.i. with [^18^F]FDG-labeled NK-92-scFv(FRP5)-zeta cells confirmed higher tumor accumulation if compared with non-modified-NK92 radiolabeled cells. Histopathological findings confirmed the accumulation of the NK-92-scFv(FRP5)-zeta cells and the absence of NK-92 cell. For this study, the QUADAS-2 analysis reported a bias concerning the unspecified origin of cell lines.

### 3.5. Future Perspectives

In recent years, understanding of NK cell potential generated high expectations and many efforts have been made to develop new immunotherapies. Unfortunately, as for other immunotherapeutic approaches, the lack of appropriate diagnostic tools to predict potential responders and evaluate therapy efficacy is still an open challenge. Molecular imaging plays an important role in tracking autologous cells in vivo, but no technique for imaging NK cell trafficking has been able to enter in the clinical practice for different reasons. An ideal technique should allow in vivo, non-invasive visualization of cells without toxicity or altered cell biodistribution. From this point of view, the ex vivo labeling approaches seem less promising due to the need to manipulate cells before reinfusion with possible occurrence of signal dilution over time. Moreover, the low number of NK cells that can be purified from peripheral blood of a patient is another important limitation for both in vitro labeling with radiopharmaceuticals or contrast agents [84]. Therefore, the best strategy to image NK cell trafficking in vivo is the in vivo targeting with radiolabeled probes, because nuclear medicine imaging has higher sensitivity and tissue penetration if compared to OI or MRI. Moreover, the parallelism with T cell-based therapies and imaging reveals that prediction of success and follow-up of immunotherapies can be achieved by using in vivo targeting strategy with several radiolabeled probes (monoclonal antibodies, proteins or peptides) [85,86]. To reach this goal, a better understanding of NK cell biology is needed to find new specific biomarkers that can help researchers in the development of more specific labeled probes. Small peptides would be particularly promising, since they can be easily radiolabeled, even at high temperatures, with short-lived isotopes like gallium-68 or fluorine-18 for PET applications. This can be translated in lower radiation burden, higher resolution and possibility to perform quantitative analyses. Regardless of which imaging technique will prove to be superior in future, the availability of a proper diagnostic tool to in vivo track NK cells will drastically impact cancer patient management.

Finally, as CAR-T and CAR-NK cells are becoming a real therapeutic alternative for many cancer types, evaluation of treatment response must be as much precise as possible. Therefore, in addition to molecular imaging strategies, new in vitro companion diagnostics (CDx) should be implemented, when possible. These consist in specific assays, as liquid or tissues biopsies, immunoassays, genetic tests and others, that can help in characterize the biological status of each patient to help physician in choosing the best therapeutic approach [87]. As a matter of facts radiomics is more and more associated to genomics, metabolomics and other CDx for the complete characterization of patients and personalized treatment. Relying on a single biomarker for therapy decision making may lead to underwhelming results in some patients with advanced cancers. Indeed, preliminary results of clinical trials revealed that tests like the PD-L1 IHC 22C3 pharmDx are not always indicative of the expression status of a specific biomarker [88]. This is mainly due to tumor heterogeneity that leads to dynamic changes in PD1 expression across different metastases. The recent FDA approval of the combination therapy with nivolumab and pembrolizumab, together with a specific diagnostic assay, demonstrated the contribution of these tests in maximizing efficacy and reducing side effects [89]. In general, assays that require a biopsy are less suitable in multimetastatic patients because some lesions might not be accessible. Blood tests may overcome this limitation, as reported by Sottile et al. They demonstrated that before anti-CTLA4 therapy, patients affected by malignant mesothelioma showed reduced frequencies of CD56dim and increased frequencies of CD56bright NK cells [90]. After therapy, the ratio reverted back to normal physiological conditions.

Therefore, the introduction of a CDx similar to the nCounter® CAR-T Characterization Panel specific for CAR-NK cells could be a welcome addition as it could be helpful in assessing the purity and functionality of NK cells at any stage. Unfortunately, this will not solve the problems related to tumor heterogeneity. Only in vivo monitoring of NK cells can give reliable information about tumor infiltration burden of adoptive cell transfer NK cells therapies.

We believe that the issues raised in the present review partially explain the lack of any open clinical trial to evaluate imaging of NK cells in humans, but we foresee that upcoming technical improvements and the implementation of new in vitro assays will allow us to overcome these limitations.

## 4. Conclusions

This review, on NK cell imaging, shows a very heterogeneous scenario with either poorly standardized studies or unclear experimental designs.

OI studies, on NK cell imaging, proved to be well designed and give the idea of a good applicability for drug evaluation and therapy follow-up. Unfortunately, the low number of available studies represents a major limitation together with low tissue penetration of fluorescence. Therefore, at present, this approach is a very good option in a preclinical setting, but far from reaching the clinical routine.

MRI is reported in a higher number of studies, mainly conducted with ex vivo SPION-labeled NK cells, but none of them were performed in humans. Moreover, the investigated diseases were very heterogeneous to be appropriately compared.

As far as nuclear medicine imaging is concerned, there are four reports of human studies performed with ex vivo labeled NK cells with ^111^In-oxine. More reports are present on animal studies by both ex vivo and in vivo NK cell labeling. Unfortunately, most of these papers have potential sources of bias, mainly due to patient/animal model selection. Indirect, in vivo, approaches with radiolabeled monoclonal antibodies seem to be the best alternative so far, but require the development of humanized or F(ab)/F(ab)2 antibodies to be tested in humans without inducing the production of human anti-mouse antibodies (HAMA).

## Figures and Tables

**Table 1:** Advantages and disadvantages of different imaging modalities.

Heading	Modality	Advantages	Disadvantages	References
**Optical imaging**	MI	Visualization of NK cell processes and interactions; time-lapse imaging	Photobleaching, phototoxicity; diffraction limit of light; no translational potential for in-vivo imaging	[48,49,50,51,52]
	BLI	Short acquisition time; high sensitivity (100 cells); high signal-to-noise ratio (no background signal)	No translational potential for in-vivo imaging; diffraction and absorption of light by tissues; immunogenicity or gene silencing; erroneous readouts; signal quantification; half-life and stability of enzymes; limited penetration depth (3 cm)	[44,45]
	FLI	Low cost; radiation free; easy labeling method; real-time	Tissue autofluorescence and absorption; limited penetration depth (1 cm); poor spatial resolution (photon scattering); limited quantification	[46,47]
**Nuclear medicine imaging**	PET	High sensitivity and specificity; no depth limit; clinically applicable; quantitative	Ionizing radiation exposure; expensive; relatively low spatial resolution 5 mm; no standardized NK cell labeling method	[55,56,57]
	SPECT	High sensitivity and specificity; no depth limit; clinically applicable; cell tracking at late time points	Ionizing radiation exposure; expensive; long scan times; relatively low spatial resolution 10 mm; no standardized NK cell labeling method	[55,56,57]
**Magnetic Resonance Imaging**	MRI	High resolution (more than 0.1 mm); no ionizing radiation exposure; clinically applicable; possible quantification (indirect)	Lower sensitivity than PET/SPECT; high costs; contrast agents interference with NK cells; long scan times	[53,54]

MI = Microscopy; BLI = Bioluminescence Imaging; FLI = Fluorescence Imaging; PET = Positron Emission Tomography; SPECT = Single Photon Emission Tomography; MRI = Magnetic Resonance Imaging.

**Table 2:** Summary of papers included in the review.

Technique	Paper [Ref.]	Modality	Cell type	Labeling agent	Subject	Disease/Model	Purpose
*Optical*	Tavri, S., 2009 [61]	OI	NK-92/NK-92-scFv(MOC31)-zeta	DiD (C67H103CIN2O3S; 1,19-dioctadecyl-3,3,39,39 tetramethylindodicarbocyanine)	Rat	Prostate cancer	Tracking
	Lim, Y.T., 2009 [62]	OI	NK-92MI	CD56 Ab-Quantum Dots (QD705)	Mouse	Malignant melanoma	Tumor targeting and therapeutic efficacy
	Jang, E., 2012 [63]	MRI	NK-92MI	Fe_3_O_4_/SiO_2_ nanoparticles	Mouse	B cell lymphoma	Tumor targeting
	Uong, T.N.T., 2018 [64]	OI	Human NK	ESNF13	Mouse	Triple-negative breast cancer	Real time tracking and tumor targeting
	Lee, J., 2019 [65]	OI	Human NK	DiR (1,1′-Dioctadecyl-3,3,3′,3′-Tetramethylindotricarbocyanine Iodide)	Mouse	Pancreatic ductal adenocarcinoma	Efficacy of NK-cell -recruiting protein-conjugated antibody (NRP-body)
*Magnetic Resonance*	Meier, R., 2011 [66]	MRI	NK-92/NK-92-scFv(MOC31)-zeta	Ferumoxides	Mouse	Prostate cancer	Tumor targeting
	Mallett, C.L., 2012 [67]	MRI	KHYG-1	MoldayION Rhodamine B	Mouse	Prostate cancer	Tumor targeting with intravenous, intraperitoneal, subcutaneous injection
	Sheu, A.Y., 2013 [68]	MRI	NK-92MI	SPION	Rat	Hepatocellular carcinoma	Tumor targeting
	Daldrup-Link, H.E., 2005 [69]	MRI	NK-92/NK-92-scFv(FRP5)-zeta	Ferumoxides/Ferucarbotran	Mouse	HER2/neu-positive mammary tumors	Tumor targeting
	Li K., 2015 [70]	MRI	NK-92MI	Heparin-protamine-ferumoxytol nancomplex	Rat	Hepatocellular carcinoma	Tracking and tumor targeting
	Somanchi, S.S., 2016 [71]	MRI	Human NK	fluorine-19	Mouse	Brain tumor	Biodistribution, homing and persistence
	Bouchlaka, M.N., 2016 [72]	MRI	Human NK	fluorine-19	Mouse	Neuroblastoma, melanoma and B cell lymphoma	Tracking and tumor targeting
	Su, Z., 2018 [73]	MRI	Mouse NK (LNK)	Ferumoxytol (Feraheme®)	Rat	Hepatocellular carcinoma	Tumor targeting and therapeutic efficacy
*Nuclear Medicine*	Hercend, T., 1990 [74]	NM (γ- camera)	Lymphokine-activated natural killer (LANAK)	^111^In-oxine	Human	Metastatic renal cell cancer	Biodistribution
	Brand, J.M., 2004 [75]	NM (γ- camera)	Allogenic human NK	^111^In-oxine	Human	Metastatic renal cell cancer	Biodistribution and tumor targeting
	Meller, B., 2004 [76]	NM (γ- camera)	Allogenic human NK	^111^In-oxine	Human	Metastatic renal cell cancer	Tumor targeting
	Matera, L., 2006 [77]	NM (γ- camera)	Adherent NK cell (A-NK)	^111^In-oxine	Human	Colon carcinoma	Difference in tumor targeting with intravenous or intra-arterial injection
	Galli, F., 2015 [78]	NM (γ- camera)	Allogenic human NK	^99m^Tc-anti-CD56 mAb	Mouse	Anaplastic thyroid cancer	Tracking and tumor targeting
	Melder, R.J., 1993 [79]	NM (PET)	Murine activated NK (ANK)	[^11^C]methyl iodide	Mouse	FSaII Fibrosarcoma	Biodistribution and tumor targeting
	Melder, R.J., 1994 [80]	NM (PET)	Murine activated NK (ANK)	[^18^F]FDG; [^11^C]methyl iodide	Mouse	None	Biodistribution
	Meier, R., 2008 [81]	NM (PET)	NK-92/NK-92-scFv(FRP5)-zeta	[^18^F]FDG	Mouse	HER2/neu-positive mammary tumors	Tracking

OI = Optical Imaging; MRI = Magnetic Resonance Imaging; NM = Nuclear Medicine; SPION = iron-oxide nanoparticles; PET = Positron Emission Tomography.

**Table 3:** Summary of Quality Assessment of Diagnostic Accuracy Studies (QUADAS-2) analysis.

Technique	Paper	Risk of Bias				Applicability Concerns		
		Subject Selection	Index Test	Reference Standard	Flow and Timing	Subject Selection	Index Test	Reference Standard
*Optical*	Tavri, S., 2009 [61]							
	Lim, Y.T., 2009 [62]							
	Jang, E.S., 2012 [63]							
	Uong, T.N.T., 2018 [64]							
	Lee, J., 2019 [65]							
*Magnetic Resonance*	Meier, R., 2011 [66]							
	Mallett, C.L., 2012 [67]							
	Sheu, A.Y., 2013 [68]							
	Daldrup-Link, H.E., 2005 [69]							
	Li, K., 2015 [70]							
	Somanchi, S.S., 2016 [71]							
	Bouchlaka, M.N., 2016 [72]							
	Su, Z., 2018 [73]							
*Nuclear Medicine*	Hercend, T., 1990 [74]							
	Brand, J.M., 2004 [75]							
	Meller, B., 2004 [76]							
	Matera, L., 2006 [77]							
	Galli, F., 2015 [78]							
	Melder, R.J., 1993 [79]							
	Melder, R.J., 1994 [80]							
	Meier, R., 2008 [81]							

Risk of bias: green = low; red = high; yellow = unclear.

## Appendix A

**Table A1:** QUADAS-2 questionnaire for evaluation of included studies.

Animals/Patients selection		Index test				Reference standard	Flow and timing	
Was a consecutive or random sample patients enrolled?	Were the inclusion/exclusions criteria properly defined?	Were NK cells properly isolated?	Can the labeling method synthesis be a source of bias? (QCs, SA)	Were the index test results interpreted without knowledge of the results of the reference standard?	Were further in vitro, ex vivo tests performed to support main results?	Is the used reference standard appropriate for the study?	Was there an appropriate interval between index tests and reference standard?	Did all patients receive a reference standard?
Could the selection of patients have introduced bias?		Could the conduct or interpretation of the index test have introduced bias?				Could the reference standard or its interpretation have introduced bias?	Did all patients receive the same reference standard?	

QCs = Quality Controls; SA = Specific Activity.
